# The FREGAT biobank: a clinico-biological database dedicated to esophageal and gastric cancers

**DOI:** 10.1186/s12885-018-3991-8

**Published:** 2018-02-06

**Authors:** Christophe Mariette, Florence Renaud, Guillaume Piessen, Patrick Gele, Marie-Christine Copin, Emmanuelle Leteurtre, Christine Delaeter, Malek Dib, Stéphanie Clisant, Valentin Harter, Franck Bonnetain, Alain Duhamel, Véronique Christophe, Antoine Adenis, Fregat Working Group, Olivier Glehen, Nicolas Carrere, Jacques Jougon, Denis Collet, François Paye, Denis Pezet, Bernard Meunier, Frédéric Di Fiore, Xavier Benoît D’journo, Olivier Bouché, Marie-Pierre Galais, Gil Lebreton, Nicolas Regenet, Frédéric Borie, Diane Goere, Jean-Michel Fabre, Emmanuelle Samalin, Jean Marc Sabate, Christophe Penna, Marc Pocard, Jack Porcheron, Olivier Tiffet, Cécile Brigand, Jean-Marc Regimbeau, Muriel Mathonnet, Pierre-Yves Brichon, Simon Msika, Adeline Germain, Thierry Conroy, Côme Lepage, Philippe Maingon, Jean-Philippe Metges, Medhi Ouaissi, Anne Berger, Frédérique Peschaud, Eric François, Jérôme Mouroux, Bertrand Dousset, Jean Marc Gornet, Brice Paquette, Jean-Christophe Vaillant, Janick Selves, Alexandra Traverse-Glehen, Genevieve Belleannée, Jean-François Fléjou, Pierre Dechelotte, Bruno Turlin, Christèle Moulin, Dominique Figarella-Branger, Doriane Barets, Marie-Danièle Diebold, Frederic Bibeau, Cécile Blanc Fournier, Jean-François Mosnier, Pascal Roger, Jean-Yves Scoazec, Patrick Bruneval, Valerie Rigau, Antoine Martin, Catherine Guettier, Rachid Kaci, Michel Péoc’h, Marie-Pierre Chenard, Henri Sevestre, François Labrousse, Marie-Hélène Laverriere, Maggy Grossin, Jacqueline Champigneulle, Agnès Leroux, Alain Bonnin, Marie-Cécile Nicot, Jean-François Emile, Natacha Chereau, Paul Hofman, Jean-François Michiels, Serge Guyétant, Benoît Terris, Philippe Bertheau, Severine Valmary-Degano, Blandine Massemin, Wacrenier Agnès, Crinquette Marie

**Affiliations:** 1 Department of Digestive and Oncological Surgery, Univ. Lille, Claude Huriez University Hospital, Place de Verdun, 59037 Lille, Cedex France; 2 Univ. Lille, UMR-S 1172 - JPArc - Centre de Recherche Jean-Pierre AUBERT Neurosciences et Cancer, F-59000 Lille, France; 3 Inserm, UMR-S 1172, F-59000 Lille, France; 4 SIRIC OncoLille, Lille, France; 5 Department of Pathology, Centre de Biologie Pathologie, Univ.Lille, University Hospital, F-59000 Lille, France; 6 Centre de Ressources Biologiques/CIC1403, Univ.Lille, University Hospital, F-59000 Lille, France; 7 Maison Régionale de la Recherche Clinique, Univ. Lille, F-59000 Lille, France; 8 Unité Intégrée de Recherche Clinique, Oscar Lambret Center, Univ. Lille, F-59000 Lille, France; 9 Centre de Traitement des Données du Cancéropôle Nord-Ouest, Centre François Baclesse, F-14076 Caen, France; 10 Methodology and Quality of Life in Oncology Unit, University Hospital of Besançon, Besançon, France; 11 EA 3181 University of Franche-Comté, Besançon, France; 12 Department of Biostatistics, Univ. Lille, University Hospital, F-59000 Lille, France; 13 CNRS, CHU Lille, UMR 9193 - SCALab, Sciences Cognitives et Sciences Affectives, Univ. Lille, F-59000 Lille, France; 14 Department of Gastrointestinal Oncology, Oscar Lambret Center, Univ. Lille, F-59000 Lille, France

**Keywords:** Esophageal cancer, Gastric cancer, Biobank, Clinico-biological database, Epidemiology, Quality of life, Human and social sciences, Research, FREGAT

## Abstract

**Background:**

While the incidence of esophageal and gastric cancers is increasing, the prognosis of these cancers remains bleak. Endoscopy and surgery are the standard treatments for localized tumors, but multimodal treatments, associated chemotherapy, targeted therapies, immunotherapy, radiotherapy, and surgery are needed for the vast majority of patients who present with locally advanced or metastatic disease at diagnosis. Although survival has improved, most patients still present with advanced disease at diagnosis. In addition, most patients exhibit a poor or incomplete response to treatment, experience early recurrence and have an impaired quality of life. Compared with several other cancers, the therapeutic approach is not personalized, and research is much less developed. It is, therefore, urgent to hasten the development of research protocols, and consequently, develop a large, ambitious and innovative tool through which future scientific questions may be answered. This research must be patient-related so that rapid feedback to the bedside is achieved and should aim to identify clinical-, biological- and tumor-related factors that are associated with treatment resistance. Finally, this research should also seek to explain epidemiological and social facets of disease behavior.

**Methods:**

The prospective FREGAT database, established by the French National Cancer Institute, is focused on adult patients with carcinomas of the esophagus and stomach and on whatever might be the tumor stage or therapeutic strategy. The database includes epidemiological, clinical, and tumor characteristics data as well as follow-up, human and social sciences quality of life data, along with a tumor and serum bank.

**Discussion:**

This innovative method of research will allow for the banking of millions of data for the development of excellent basic, translational and clinical research programs for esophageal and gastric cancer. This will ultimately improve general knowledge of these diseases, therapeutic strategies and patient survival. This database was initially developed in France on a nationwide basis, but currently, the database is available for worldwide contributions with respect to the input of patient data or the request for data for scientific projects.

**Trial registration:**

The FREGAT database has a dedicated website (www.fregat-database.org) and is registered on the Clinicaltrials.gov site, number NCT 02526095, since August 8, 2015.

## Background

In 2012, the worldwide incidence of esophageal and gastric cancers was estimated to be about 1,500,000 new cases (500,000 cases of esophageal and 1,000,000 of gastric cancer), with 2,110,000 new cases expected by 2025 [1]. Esophageal and gastric cancers are ranked seventh and eighth, respectively, among the causes of death from cancer, and thus, they constitute a major public health problem [2]. Recently, we have witnessed some major epidemiological changes in relation to esophageal and gastric cancers, as follows: (i) a significant increase in the incidence of adenocarcinomas of the esophagus and the esophagogastric junction in the Western world, (ii) a reduction in the incidence of distal gastric cancers that occur in parallel with an increase in the incidence of proximal gastric cancers and tumors of the esophagogastric junction, and finally (iii) an increase in the incidence of adenocarcinomas with signet ring cells [2, 3]. In spite of this high incidence of esophageal and gastric cancers and the recent major epidemiological changes, much less research is performed on these types of carcinomas compared with other cancer types. It is, therefore, vital to intensify the research of both esophageal and gastric cancers.

Treatment that is aimed to cure esophageal and gastric cancers usually involves surgical excision, except for certain locally advanced types of esophageal cancer, which can be treated exclusively with radio(chemo)therapy [4, 5]. The risks of early and late relapse following surgery alone have led to the design of multiple, complex treatment strategies, including a combination of surgery, endoscopy, chemotherapy, radiotherapy and immunotherapy. At the unresectable and/or metastatic stage, numerous treatment options also exist that aim to improve survival and quality of life, while minimizing the toxic effects associated with treatment. Currently, the proposed approaches are global and are rarely individualized to target sub-groups of at-risk patients.

Despite this range of treatment strategies, the prognosis for esophageal and gastric cancers remains particularly bleak, as the 5-year overall survival for all stages is approximately 10–15% for esophageal cancer and 25% for gastric cancer [6, 7]. These diseases will persist and/or recur in most patients because, during the course of treatment, they display an intrinsic or acquired resistance to the anti-tumor therapies implemented, which results in impaired survival and quality of life. Currently, the mechanisms associated with this resistance to treatment are poorly understood and are multifactorial. These mechanisms involve clinical and biological factors associated with the host and the tumor and possibly the patient’s psycho-social environment. Consequently, a study of the mechanisms of resistance in esophageal and gastric cancers requires the use of a prospective database dedicated to patients with esophageal or gastric cancers that contains medical and surgical clinical data as well as epidemiological, psychological, emotional, social and biological data (collection of blood and tumor samples): the FREGAT clinico-biological database (FREGAT CBD).

## Methods/design

### FREGAT network

In 2010, a European network was established that aimed to share the efforts of various French and French-speaking European teams in relation to esophageal and gastric cancer research. This network, known as FREGAT (French Research in Esophageal and Gastric Tumors) working group, is successfully managing various research projects, as follows:

- Through retrospective cohorts, often driven in partnership with other scientific societies (FRENCH, Fédération de Recherche en Chirurgie (*French Surgical Research Federation*), AFC Association Française de Chirurgie (*French Surgical Association))*, numerous articles were published in journals with high impact factors;
- National and European prospective and randomized studies;
- A contribution to European research projects.

This sustained activity and the visibility of the FREGAT network meant that, in 2012, funding was obtained from the Institut National du Cancer (*National Cancer Institute*) for FREGAT CBD, the only worldwide project of this scale; this allowed research on esophageal and gastric cancers to be approached from an innovative angle. Unlike randomized trials, which attempt to answer a question asked a priori following what is often a long inclusion period of patient selection, the CBD-type approach allows for wide-ranging and prospective collection of information about non-selected consecutive patients. This results in a response to a scientific query of interest. The very large sample size means that investigators can control for any potential bias. This new approach is a way to accelerate research, and at the same time, reduce its cost.

### The FREGAT clinico-biological database

The FREGAT CBD is registered as Bio-Medical Research excluding health products with the Agence Nationale de Sécurité du Médicament et des produits de santé (*French Medical Products Agency*) and has a dedicated internet site (https://www.fregat-database.org/). The FREGAT CBD aims to enroll a mean of 500 patients per year. Inclusions began in early 2015, and each patient is followed-up for 3 years.

This research comprises the following:

- The recruitment of all newly diagnosed patients who were completely treatment-naive for resectable or unresectable esophageal or gastric carcinoma (whatever the histological type, tumor stage and treatment strategy), received treatment at participating centers;
- A collection of tumor samples (pre-therapeutic biopsies, post-therapeutic biopsies, resected specimens) in compliance with current quality charters. These samples are stored at the relevant investigator center and are ideally frozen, although failing this, storage in paraffin wax is acceptable;
- The collection of blood samples has now been established at 6 centers where a great deal of recruitment has occurred and where a Biological Resources Center is located (Lille, Montpellier, Bordeaux, Marseille, Lyon, Rennes). This selection, basically linked to the available budget, can be adapted in the future depending on accessible financial resources and the dynamism of the selected centers. As is customary, these designated Biological Resources Centers will be responsible for the quality control of the samples;
- An epidemiological and socio-economic database;
- Questionnaires related to Human and Social Sciences;
- Questionnaires on Quality of Life.

The questionnaires on Human and Social Sciences and Quality of Life are given to every patient by the investigator at each center and are identified by a unique patient number. They are then collected by the sponsor who is responsible for entering the data in the electronic case report form (e-CRF).

### Study population

All patients who are completely treatment-naive with newly diagnosed esophageal or gastric carcinoma at a participating center are included after consent has been accepted and signed, whether they have undergone surgery, and no matter their tumor histological type, tumor stage and treatment strategy.

#### Criteria for inclusion

- Patients with carcinoma of the esophagus, esophagogastric junction or stomach, those who were newly diagnosed by biopsy, no matter the cancer subtype, tumor stage or envisaged treatment.
- Man or woman ≥ 18 years of age.
- Treatment-naive in relation to these particular cancers.
- Covered by the social welfare scheme.
- Patients who voluntarily signed an informed consent form for retrieval of blood samples, completion of questionnaires and collection of patient information.

Failing this, a patient who has received neoadjuvant treatment may be included in the FREGAT CBD, but this is subject to the recovery of pre-treatment data and whether all possible efforts have been made to access the tumor samples (tumor blocks) generated prior to any treatment. Patients who have participated in a clinical trial may be included in the FREGAT CBD. There is no exclusion period.

#### Criteria for exclusion

- Man or woman aged < 18 years.
- Person deprived of liberty or under trusteeship (including guardianship).
- Adult person incapable of expressing his or her consent.
- Patient already included in the FREGAT CBD.
- Patient refusal.

### Aims of the research

The formalization of the database as a bio-medical research project has led to a number of defined and stated aims, but other research aims may be approached via scientific research projects.

#### Primary aim

To identify the clinical, biological and tumor variables associated with 3-year relapse in patients who are treated for stage I to IV esophageal or gastric cancer, through the establishment of a prospective, multicenter, clinico-biological database.

#### Secondary aims

- To evaluate the impact of the range of usual treatment strategies on 3-year relapse, 3-year survival and health related quality of life;
- To identify the treatment related factors associated with the 3-year relapse to identify the most effective and the least toxic treatment combinations;
- To describe the individual, social and behavioral characteristics;
- To identify the individual and collective determining factors that influence the times for access to care and the start of treatment;
- To identify new prognostic factors and those that predict 3-year relapse.

### Statistical plan

The sample size should be large enough to allow detection of factors related to 3-year relapse, whatever could be the disease stage, the tumoral location or the therapeutic strategy. Because of the nature of the study, being a prospective database, our aim is to include a large number of gastric and esophageal cancer patients to observe enough events allowing us to identify a large panel of variables associated with tumoral relapse at 3 years. The estimated inclusion rate of 500 new cases per year of esophageal and gastric cancer at a national level is estimated to be feasible based on the national incidence of esophageal and gastric seen in France per year in participating centers. With a 10-year inclusion period, a sample size of 5000 patients will be available, based on the following assumptions: X% of gastric and Y% of esophageal cancer patients will be included in the database over the study period, with P% of patients expected to be lost of follow-up and/or with not analyzable data. If we consider N, the smallest group of patients and a 5-year recurrence rate at R% in this group, we will have Z events, allowing us to analyse V predictive factors. As an example, with a *P* value of 20%, X = 65% and *R* = 90% in the gastric cancer group and Y = 35% and *R* = 75% in the esophageal cancer group, considering the smallest group (Y in the present example) will allow us to analyse the following number of events Z = 5000*.8*.35*.75 = 1050. The number of variables linked to recurrence analysable will be consequently V = 1050/20 = 52,5.

### Variables collected

Clinical, biological and epidemiological data and the treatment characteristics of the patients are collected in the e-CRF (https://fregat.ctd-cno.org/CSOnline/).

The variables collected are described below:

- Epidemiological: month, year and place of birth, sex, address.
- Clinical: WHO status, weight, height, comorbidities, date of first symptom, date of first consultation with a general practitioner and with a specialist, type of specialist consulted, risk factors, nutritional support, location of the tumor, cTNM stage, presence of other cancers.
- Biological: presence of albuminemia.
- Therapeutic: treatment strategy initially indicated, treatment given, chemotherapy, surgery, radiotherapy, evaluation of response and tolerance to treatment.
- Human and social sciences: data from the PEC and CARE questionnaires together with a visual analogue scale, HADS, MOS-SSS, and quality of life (QLQ-C30 and QLQ-OG25 questionnaires).
- Pathological: histological type, tumor differentiation, Lauren and WHO classification, radical nature of the surgery, (y)pTNM staging, and histological response to neoadjuvant treatment. Qualification of tumor samples: type and number of samples, distribution of samples for freezing and/or paraffin-embedding, and the evaluation of the quantity of tumor cells in cryopreserved and fixed tissues.
- Blood samples: type (EDTA, heparinized plasma, buffy coat, serum) and number of samples.
- Quality control of samples: time interval between resection of the tumor and when it is given to the pathologist, method of preservation, macroscopic examination, whether the fragment was frozen/embedded in paraffin, and storage (number of samples, identification).
- Follow-up: death (date and cause), follow-up duration, recurrence or relapse.
- The information gathered in the database extends from the point when the clinical follow-up of the patients begins, with the usual visits and follow-up methods, at each investigator center. The information must be updated on an annual basis. In addition, the information must be updated when an event such as relapse or death occurs. Finally, this list of variables, which have been established a priori, can be amended to reflect the research projects initiated by the scientific committee once these projects have been validated.

### How the research is conducted in practice

After the criteria are verified for inclusion and non-inclusion, the investigator provides the patient with information and gathers two copies of his or her signed consent *(*https://fregat.ctd-cno.org/CSOnline/*).* To proceed to inclusion, after the consent is signed, the investigator completes the inclusion form on the e-CRF to obtain the unique FREGAT CBD inclusion number, which is automatically generated by the Data Processing Centre and is transcribed on all the FREGAT CBD documents and questionnaires. This will be the only way to identify a patient.

**Fig. 1:**
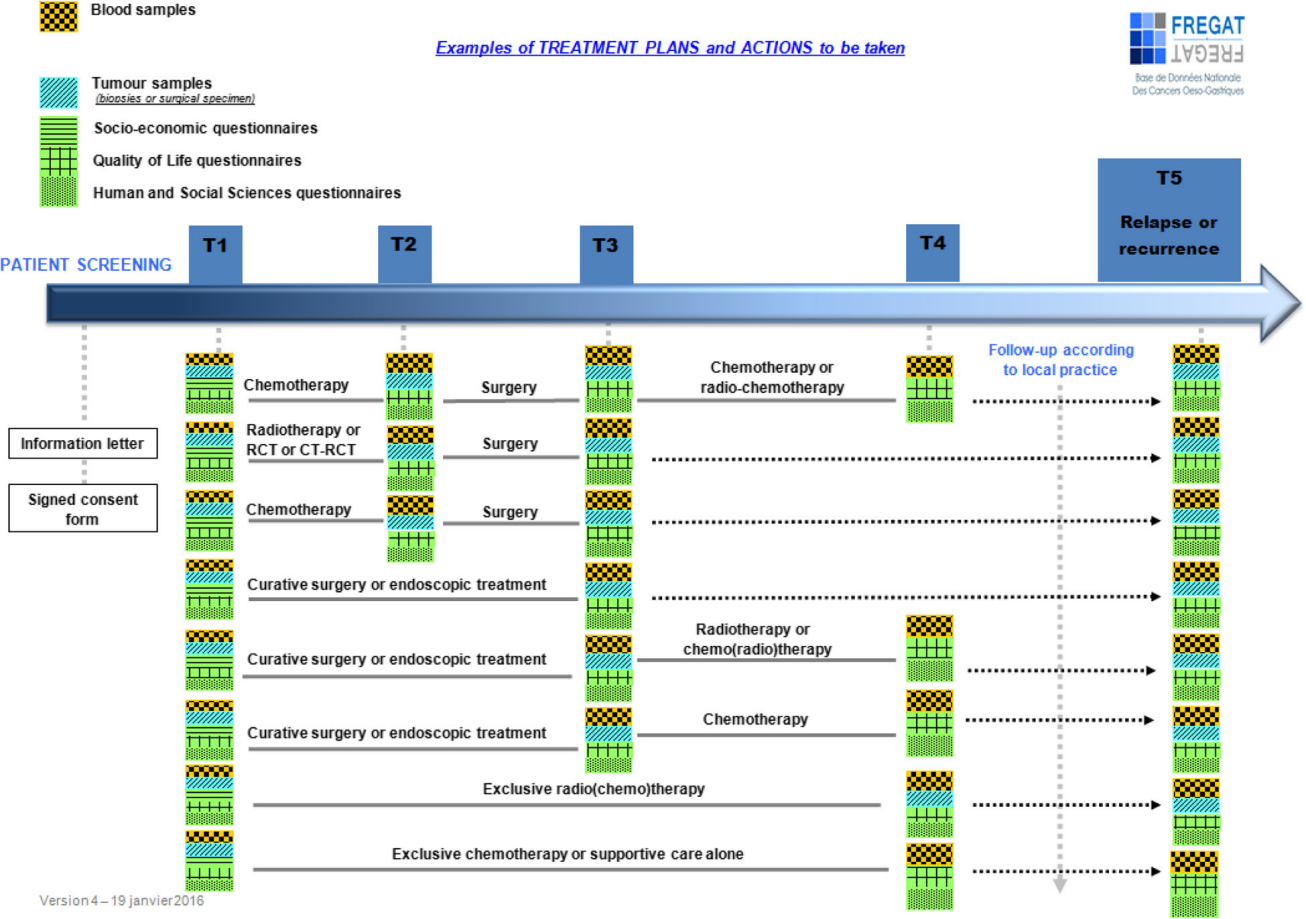
Examples of the treatment plans for esophageal or gastric cancer and actions to be taken for the FREGAT research

The frequency of examinations and the point when the self-assessment questionnaires are submitted depends on the patient’s treatment regimen, as described in Fig. 1.

- ***T1: On inclusion***

These examinations must be performed once a patient is included, when the questionnaires are submitted, or at the very latest, on the date the first treatment is administered (surgery, chemo(radio)therapy).

**Clinical examination:** Clinical summary (WHO status, weight, height), clinical diagnosis (location of the tumor, cTNM classification, first histological diagnosis (date, type, extent of tumor differentiation).

**Fig. 2:**
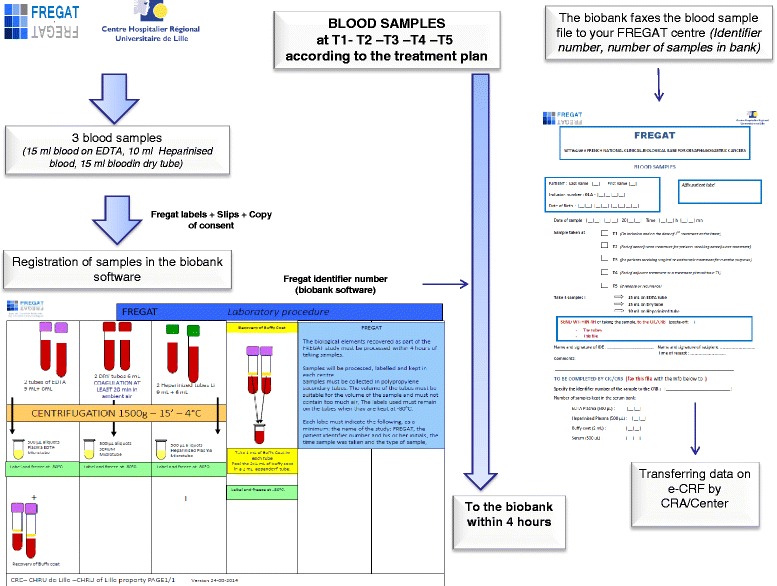
Process for the collection and banking of blood samples

**Blood samples:**  Three blood samples (15 mL in an EDTA tube, 15 mL in a dry tube, 10 mL in a heparinized tube) are obtained at the time of inclusion at eligible centers and are stored at the Biological Resources Center at the relevant investigator center (Fig. 2).

**Fig. 3:**
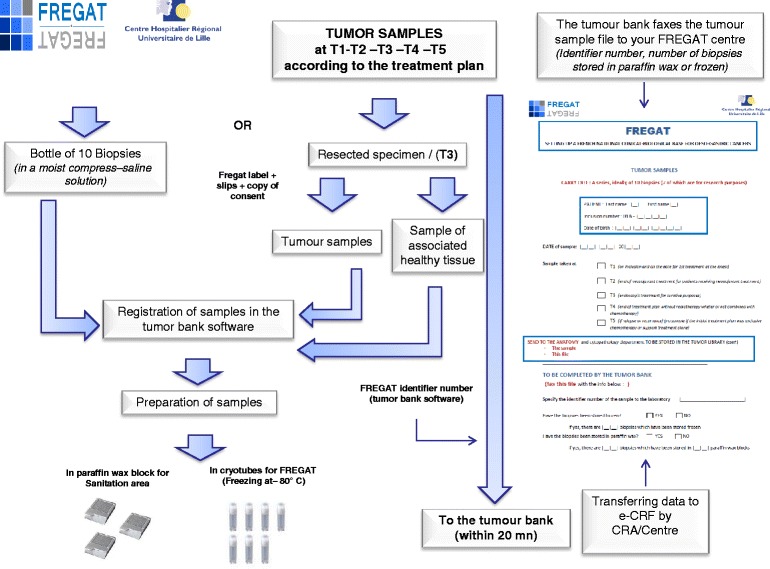
Process for the collection and banking of tumor samples

**Tumor samples:**  A series of samples, ideally involving 10 pre-treatment biopsies (7 for research purposes), is obtained to confirm the presence of esophageal or gastric cancer. These samples are stored at the relevant investigator center and are preferably frozen; however, preservation in paraffin wax is also acceptable (Fig. 3).

**Questionnaires**  - Socio-economic (social situation, professional activity, lifestyle).

- Human and Social Sciences (PEC, CARE, MOS-SSS, HADS).

- Quality of Life (QLQC30, QLQOG25).

- ***T2: End of neoadjuvant treatment***

T2 is only applicable for patients who receive neoadjuvant treatment. These examinations are performed, and the questionnaires are submitted on the date of the final treatment or within 2 weeks following chemo(radio)therapy.

**Clinical examination:** WHO status, weight.

**Treatment regimen:** List of antitumor treatments and occurrence of grade 3–4 severe toxicities.

**Blood samples:**  Three blood samples (15 mL in an EDTA tube, 15 mL in a dry tube, 10 mL in a heparinized tube) are obtained at eligible centers and are stored at the Biological Resources Center at the relevant investigator center (Fig. 2).

**Questionnaires**:

- Human and Social Sciences (PEC, CARE, MOS-SSS, HADS).

- Quality of Life (QLQC30, QLQOG25).

- ***T3: Surgery or endoscopic treatment for curative purposes***

T3 is only applicable for patients who receive surgical or endoscopic treatment for curative purposes.

**Collection:** Details about the intervention, any post-operative complications, and pathologic data.

**Blood samples:**  Three blood samples (15 mL in an EDTA tube, 15 mL in a dry tube, 10 mL in a heparinized tube) are obtained at eligible centers and are stored at the Biological Resources Center at the relevant investigator center (Fig. 2).

**Tumor samples:**  Tumor samples stored at the relevant investigator center, preferably frozen, but preservation in paraffin wax is also accepted (Fig. 3).

Questionnaires are distributed during the post-operative stage within 30 days of the intervention.

**Questionnaires**:

- Human and Social Sciences (PEC, CARE, MOS-SSS, HADS).

- Quality of Life (QLQC30, QLQOG25).

- ***T4: End of adjuvant treatment. T4 is only applicable for patients receiving adhuvant treatment***

These examinations are performed, and the questionnaires are distributed on the date of the final treatment or within 2 weeks following chemo(radio)therapy.

**Clinical examination:** WHO status, weight.

**Treatment regimen**: List of antitumor treatments and occurrence of grade 3–4 severe toxicities.

**Blood samples:**  Three blood samples (15 mL in an EDTA tube, 15 mL in a dry tube, 10 mL in a heparinized tube) are obtained at eligible centers and are stored at the Biological Resources Center at the relevant investigator center (Fig. 2).

**Tumor samples:** In the event of radiotherapy or exclusive chemo(radio)therapy.

 A series of samples, ideally consisting of 10 biopsies (7 for research purposes) is obtained and stored at the relevant investigator center and are preferably frozen; however, preservation in paraffin wax is also accepted (Fig. 3).

**Questionnaires:**

- Human and Social Sciences (PEC, CARE, MOS-SSS, HADS).

- Quality of Life (QLQC30, QLQOG25).

- ***Follow-up for 3 years:*** Follow-up is performed according to the practices applied at each center. The latest data regarding the patient are collected every year at a minimum and are entered in the e-CRF.

- ***T5: On recurrence or relapse***

T5 is applicable to all patients.

**Clinical examination:** WHO status, weight.

**Treatment regimen:** List of antitumor treatments and occurrence of grade 3–4 severe toxicities.

**Blood samples:**  Three blood samples (15 mL in an EDTA tube, 15 mL in a dry tube, 10 mL in a heparinized tube) are obtained at eligible centers and are stored at the Biological Resources Center at the relevant investigator center (Fig. 2).

**Tumor samples:**  A series of samples, ideally consisting of 10 biopsies (7 for research purposes) is obtained and stored at the relevant investigator center, preferably frozen; however, preservation in paraffin wax is also accepted (Fig. 3).

No tumor samples are needed if only supportive care is provided.

**Questionnaires:**

- Human and Social Sciences (PEC, CARE, MOS-SSS, HADS).

- Quality of Life (QLQC30, QLQOG25).

### Collection of biological samples

The FREGAT research project links the clinical data of patients with information that pertains to the clinical, biological and tumor data. All the data contained in the FREGAT research project is gathered when the patients undergo normal treatment procedures.

The biological collection is maintained by the centers who accept the samples and by the Clinical Investigation Centre/Biological Resources Center at the corresponding center. Their roles are to:

- Gather, prepare and conserve biological samples under optimum conditions to make them available to clinicians/researchers and when subsequent scientific projects are conducted.

- Identify the sample in a unique manner: the sample belongs to a particular study and patient, and thus, the sample is identified using a specific label.

- Record the sampling method and conditions, those involved in the procedure, the clinical information, any additional examinations, contamination, quality, potential danger, and the location of the sample.

- Store and manage samples with regard to aspects of referencing, storage, traceability of inputs, outputs or incidents. Storage premises must be secure.

The future use of samples shall be validated by a steering group, whose members are representative of the investigator and his/her personnel and the users of the biological collection.

These aspects of the activities performed by the Clinical Investigation Center/Center of Biological Resources are certified by the AFAQ-AFNOR to ensure that they comply with standard ISO 9001v2008 and national and European regulations on the subject of biological samples.

Consequently, they represent a major and intrinsic added value of the FREGAT CBD.

- *Collections of tumor samples (*Fig. 3*)*

Tumor samples are part of normal treatment procedures, but for the purposes of FREGAT CBD quality, we request a series, ideally consisting of 10 biopsies (7 of which are for research purposes, to be adjusted by the procedures followed by each center) to be obtained and stored at the relevant investigator center. The samples are preferably frozen, but failing this, preservation in paraffin wax is accepted. Sampling procedures occur during normal treatment times (Fig. 1).*Collection of blood samples* (Fig. 2)

A prospective biological collection is currently being established at 6 Biological Resource Centers (Lille, Montpellier, Bordeaux, Marseille, Lyon, Rennes), which were selected according to the following criteria: (i) significant volume of activity associated with cancer of the esophagus and stomach, (ii) scientific interest of the center in relation to the research topic, (iii) willingness to obtain biological samples for all patients, (iv) commitment to quality.

The elements sampled are as follows: 15 mL in an EDTA Tube, 15 mL in a dry tube, 10 mL in a heparinized tube, for a total volume of 40 mL of blood from each sampling procedure (Fig. 2).

A breakdown of the samples is presented in Table 1. The samples are processed and stored at − 80 °C at participating centers within 4 h after the samples are acquired. The storage temperature is monitored by means of continuous recordings, and the various freezers each contain a central alarm. A reserve freezer must be available to quickly compensate for any problems.

**Table 1:** Details for blood samples collection

Nature	Volume	Pre-analytical conditions	By-product	Volume of Aliquots	Storage temperature
Blood on EDTA	15 mL	Centrifugation 1500 g 15 min 4 °C	EDTA Plasma Buffy Coat Genomic DNA	500 μL 1 mL 1 mL	−80 °C
Blood on Heparin	10 mL		Heparinized plasma	500 μL	−80 °C
Blood on Dry Tube	15 mL		Serum	500 μL	−80 °C

*EDTA* Ethylene Diamine Tetraacetic Acid

### Participating teams and locations where the research is to take place

FREGAT research brings together almost all the clinical teams at the University Hospital Centres and the French Centres de Lutte Contre le Cancer (*Cancer Research Centres*), which are most likely to treat patients with these cancers. The current list of declared investigators is available on the FREGAT website (https://www.fregat-database.org/).

At present, the partners involved in the FREGAT CBD research are as follows:

- 37 participating centers, (i.e., 50 teams)
- The network of tumor banks
- The Biological Resource Centers
- The Data Processing Centers from the North-West Cancéropôle (*Cancer Research Cluster*)

It is also supported on designated platforms:

- Lille University - Human and Social Sciences platform
- Caen Epidemiology platform
- Lille University - Methodology and Biostatistics platform
- Besançon Quality of Life platform

### How should a scientific research project be filed?

The form used to file a Scientific Research Project is available on the internet site (https://www.fregat-database.org/). The completed project will be sent to proofreaders from the scientific committee, and feedback will be sent to the investigator during the month after the project was filed. The scientific relevance of the project will be evaluated, will the type of resources required and their availability. Financial support for the project to pay for the outlet and centralization of data and samples will also be considered.

### Availability to non-French centers

The first stage was to organize and establish the FREGAT CBD in France and to launch this scientific dynamic using national fund. Any interested foreign center may join the FREGAT dynamic provided it obtains the favorable opinion of the steering group and reaches a financial agreement. Any non-French team that wishes to access the data may file a Scientific Research Project; the appropriate form is available on the website https://www.fregat-database.org/ and is available in both French and English.

## Discussion

Despite efforts to optimize therapeutic strategies for better locoregional and systemic disease control, esophageal and gastric cancers still have a poor prognosis, as tumors are frequently diagnosed at an advanced stage, and there is often a delay between the appearance of initial symptoms and diagnosis. Even after multimodality treatments that combine endoscopy, surgery, chemotherapy, radiotherapy and immunotherapy are used to varying degrees, most patients experience disease persistence or recurrence related to treatment resistance of the tumor. The causes of such resistance are numerous and require urgent evaluation. In addition, the development of effective targeted treatments has been slow. The FREGAT prospective database, which is dedicated to esophageal and gastric cancers and which include tumor and serum banking, was created out of a need to identify specific epidemiological characteristics of esophageal and gastric cancer and to evaluate their impact on a given therapeutic strategy. Creation of this database was also necessary for the identification of biological, clinical and tumor paradigms that explain resistance to treatment, and the identification of social and behavioral factors that result in a delay between first symptoms and diagnosis. The availability of biospecimens is critical to epidemiologic research for the identification, development, and validation of biomarkers for cancer susceptibility, precursor states, carcinogenic exposures, and cancer progression and recurrence. Biomarkers are key in studies of the molecular pathways that lead to cancer development and progression, and they may serve as surrogate markers for drug efficacy and toxicity, and as targets for cancer prevention, diagnosis, and treatment. Personalized medicine in esophageal and gastric cancer is just beginning and needs to be further developed. Since esophageal and gastric cancers are complex cancers with multiple pathological and therapeutic drivers, resistance is common and multiple targeted therapies, which combine local and system approaches, will be required. This will rely on new clinico-epidemiological and biological studies. A comprehensive bench-to-bedside treatment-guided algorithm could provide for the optimum preoperative and/or postoperative combination of cytotoxic and targeted agents. Large specialized tumor banks and biobanks are mandatory for such projects**.**

The prospective FREGAT CBD, established by the French National Cancer Institute, relies on different well-known and currently active networks. This database is dedicated to adult patients with carcinomas of the esophagus and stomach, no matter the tumor stage or therapeutic strategy. It includes prospective data on epidemiology, clinical information, tumor characteristics, follow-up, human and social sciences and quality of life along with tumor and serum banking.

The major aims of the FREGAT database are to (i) identify new prognostic and predictive factors based on clinical, biological and histological data, (ii) analyze the molecular mechanisms of drugs to discover and develop novel therapies, (iii) validate the promising markers already identified, (iv) evaluate the impact of each therapeutic strategy and the combination of strategies at a national level in different clinical and histological subgroups of esophageal and gastric cancer patients; this would thus strengthen the personalized therapeutic approach, (v) identify epidemiological, human and social sciences determinants that may have an impact on suboptimal access to care and delays in initiation of treatment, and (vi) evaluate the impact of the therapeutic strategies on the health-related quality of life of patients.

This innovative method of research will allow for the banking of millions of data for the development of excellent basic, translational and clinical research programs in esophageal and gastric cancer to improve knowledge, therapeutic strategies and patient survival. While this database was initially developed in France on a nationwide basis, the database is now open to worldwide contributions with respect to the input of patient data or for requests for data for scientific projects.

## Abbreviations

AFAQ: Assurance Française pour la Qualité
AFC: Association Française de Chirurgie (*French Surgical Association)*
AFNOR: Association Française de Normalisation
CARE: Consultation and Relational Empathy
CBD: Clinico-biological database
e-CRF: Electronic case report form
EDTA: Ethylene Diamine Tetraacetic Acid
FREGAT: French research in esophageal and gastric tumors
FRENCH: Fédération de Recherche en Chirurgie (*French Surgical Research Federation*)
HADS: Hospital Anxiety and Depression Scale
MOS-SSS: Medical Outcomes Study-Social Support Survey
PEC: Profile of Emotional Competence
QLQ: Quality of Life
SIGAPS: Système d’Information, de Gestion et d’Analyse des Publications Scientifiques
SIGREC: Système d’Information et de Gestion de la Recherche et des Essais Cliniques
WHO: World Health Organization
